# Clinical Lectures on the Principles and Practice of Medicine

**Published:** 1867-10

**Authors:** 


Clinical Lectures on the Principles and Practice of Medicine. By JOHN HUGHES BENNETT, M.D., F.R.S.E., Prof. of the Institutes of Medicine, and Senior-Professor of Clinical Medicine in the University of Edinburgh; etc., etc., etc. Fifth American, from the Fourth London, Edition. With 575 Illustrations on wood. New York: WILLIAM WOOD & Co., 61 Walker Street. 1867.

This is a new edition of the well-known work of Bennett, on Pathology and Practical Medicine. It has been thoroughly revised by the author, and 200 or 300 pages of new matter on various subjects added, making a volume of over 1000 pages. The New York publishers have issued the American edition in good style; and the general merits of the work are too well known to the profession in this country to require extended comment.

For sale by S. C. GRIGGS & Co., 39 and 41 Lake St., and by medical booksellers generally.

